# Recent Insights on Alzheimer’s Disease Originating from Yeast Models

**DOI:** 10.3390/ijms19071947

**Published:** 2018-07-03

**Authors:** David Seynnaeve, Mara Del Vecchio, Gernot Fruhmann, Joke Verelst, Melody Cools, Jimmy Beckers, Daniel P. Mulvihill, Joris Winderickx, Vanessa Franssens

**Affiliations:** 1 Functional Biology, KU Leuven, Kasteelpark Arenberg 31, 3000 Leuven, Belgium; david.seynnaeve@kuleuven.be (D.S.); mara.delvecchio@kuleuven.be (M.D.V.); gernot.fruhmann@kuleuven.be (G.F.); joke.verelst@kuleuven.be (J.V.); melody.cools@kuleuven.be (M.C.); jimmy.beckers@student.kuleuven.be (J.B.); joris.winderickx@kuleuven.be (J.W.); 2 School of Biosciences, University of Kent, Canterbury CT2 7NJ, Kent, UK; D.P.Mulvihill@kent.ac.uk

**Keywords:** Alzheimer’s disease, yeast, Tau, amyloid β, ubiquitin, aggregation, oligomerization, prion

## Abstract

In this review article, yeast model-based research advances regarding the role of Amyloid-β (Aβ), Tau and frameshift Ubiquitin UBB^+1^ in Alzheimer’s disease (AD) are discussed. Despite having limitations with regard to intercellular and cognitive AD aspects, these models have clearly shown their added value as complementary models for the study of the molecular aspects of these proteins, including their interplay with AD-related cellular processes such as mitochondrial dysfunction and altered proteostasis. Moreover, these yeast models have also shown their importance in translational research, e.g., in compound screenings and for AD diagnostics development. In addition to well-established *Saccharomyces cerevisiae* models, new upcoming *Schizosaccharomyces pombe*, *Candida glabrata* and *Kluyveromyces lactis* yeast models for Aβ and Tau are briefly described. Finally, traditional and more innovative research methodologies, e.g., for studying protein oligomerization/aggregation, are highlighted.

## 1. Introduction

### 1.1. Alzheimer’s Disease (AD)

Alzheimer’s disease (AD) is the most common neurodegenerative disease worldwide. It accounts for approximately 60–70% of all dementia cases and affects about 6% of the population aged over 65 (late-onset AD), whereas 2–10% of patients suffer from early-onset AD [1,2]. Currently, around 50 million individuals live with this devastating chronic disease and it has been estimated that the number will increase up to approximately 106 million people by 2050 due to an increasing aging population [2,3]. At the cellular level, AD is characterized by an irreversible and progressive loss of neuronal structure and function within certain regions of the brain including the hippocampus and neocortical brain, leading to cognitive dysfunction and dementia [4]. Widespread experimental evidence also suggests that AD is characterized by synaptic dysfunction early on in the disease process, disrupting communication within neural circuits important for memory formation and other cognitive functions such as intellectuality and comprehensive capacity [5,6,7].

Therefore, damage to these brain structures results in memory loss, language difficulties and learning deficits that are typically observed within early stages of clinical manifestation of AD. In addition, upon disease progression, a decline in other cognitive domains occurs which will result in the complete inability to function independently in basic daily activities [7]. Besides AD having a profound impact on the life quality of patients, this chronic disease also imposes a huge economic burden on healthcare systems globally with an associated cost which is estimated will exceed $1 trillion by 2050 [8].

The neuronal damage is related to the accumulation of misfolded proteins into extracellular and intracellular aggregates, consisting of Aβ peptides or protein Tau, respectively [9,10]. It is not yet clear whether the presence of these two hallmarks is the cause of AD or mainly the result of a cascade of cellular events including oxidative stress, mitochondrial dysfunction and apoptosis. Either way, the exact mechanism by which these proteins damage neurons is still unknown.

### 1.2. Yeast as a Model Organism to Study AD: Advantages

Studies to gain more insights on AD primarily make use of human cell lines and transgenic mouse models. However, yeast cell models are playing an increasingly important role in unravelling the fundamental disease aspects of AD. In fact, the yeast *S. cerevisiae* is a very widely studied single-celled model organism. With more than 6000 genes distributed on 16 chromosomes, its genome was the first eukaryotic genome to be completely sequenced in 1996 [11]. Since then, it has been estimated that nearly 31% of yeast genes have human orthologues [12]. Beyond the laboratory yeast strains, many different natural, brewery and clinical isolates exist and all have a core genome of about 5000 shared genes [12]. Yeast reproduction is through mitosis of either a haploid or a diploid cell. Haploids are of 2 different mating type (a or α) and a haploid cell can only mate with a cell of the opposite mating type. Mating leads to the formation of a diploid cell that can either continue to exist and bud as a diploid or, under conditions of stress, produce spores by meiosis. Spores can then later give rise to haploid cells [12]. Haploidy implies that gene-knockout strains can easily be obtained. In 2001, a collection of isogenic yeast strains, each deleted for one of the 6000 putative open reading frames (ORFs), was created [12]. This allowed for the easy phenotypic analysis of mutants, paving the way to determining gene function. In addition, yeast cells share many conserved biological processes such as cell cycle progression, protein turnover, vesicular trafficking and signal transduction with cells of higher eukaryotes [13], including human neurons. Its short generation time (1.5 h on rich medium), means that it can be very easily cultured. Thanks to its susceptibility to simple genetic and environmental manipulations, this model organism has become a valuable tool to shed more light on the complex and fundamental intracellular mechanisms underlying neurodegenerative diseases.

So-called “humanized yeast model systems” have been constructed and used as a tool to investigate the molecular mechanisms involved in several neurological disorders [14,15]. The main advantage of using yeast is its reduced complexity compared to the mammalian models. On the contrary, Tau and Aβ have no functional yeast orthologues. Heterologous expression of Tau and Aβ can be highly informative and provides useful new insights into the pathobiology of these proteins in vivo. At the same time, yeast is an excellent screening tool for compounds that may be useful in treatment and/or prevention of AD.

### 1.3. Yeast as a Model Organism to Study AD: Limitations

Despite being a powerful and simplified model system, yeast also has its natural limitations. As a unicellular organism, the most important limitation for neurodegenerative disease research is the analysis of disease aspects that focus on multicellularity and cell–cell interactions. These interactions include synaptic transmissions, axonal transport, glial-neuronal interactions, immune and inflammatory responses and many neuronal specializations that are likely to play an important role in neurodegeneration, but cannot be recapitulated in yeast [16]. Moreover, it is also impossible to study the cognitive aspects of AD in yeast cells.

This review discusses the findings of more recent studies on neurodegenerative disorders conducted using different yeast species.

## 2. Humanized Yeast Models to Study Tau Biology

### 2.1. Protein Tau: Structure, Functions and Modifications

Protein Tau, encoded by the 16 exon long microtubule (MT)-associated protein Tau (*MAPT*) gene located on chromosome 17q21.31, is present in neuronal and glial axons, but has also been detected outside of cells [17,18,19,20,21,22]. Tau is natively unfolded and has the tendency to adapt a paperclip-like shape, in which the N- and C-terminal domains and repeat regions are all closely located to each other (see below) [23]. It is a MT associated protein, susceptive to dynamic (de-) phosphorylation. These modifications influence its main cellular function which consists of regulating MT dynamic instability i.e., the process of polymerization (rescue) and depolymerization (catastrophe) [24,25,26,27]. Besides being involved in the regulation of MT dynamics, Tau has functions in regulating axonal transport/elongation/maturation, synaptic plasticity and maintaining DNA and RNA integrity [28,29,30,31,32,33,34,35,36,37]. It is clear that Tau is involved in numerous processes and that loss of Tau function can initiate neurotoxicity through disruption of various processes in which it is involved. For a more complete overview of physiological and pathological Tau functions, we refer to [38].

A first mechanism by which Tau function is regulated is alternative splicing. Due to alternative splicing of exon 2, 3 and 10 of the *MAPT* gene, Tau can be present in 6 different isoforms differing in the number of N-terminal inserts (1 or 2) and conserved 18 amino acid long repeats (3 to 4) in the MT binding region (C-terminal region or assembly domain) [39,40]. Tau isoforms with 4 repeat regions show a stronger interaction with MT and are more efficient in MT assembly [41,42,43]. The N-terminal projection region of the protein is located adjacent to a proline rich region and has a role in MT spacing and stabilization [44,45]. In addition, it was proposed that this domain, which projects away from MT, interacts with cell organelles such as the plasma membrane, mitochondria and actin filaments [46,47,48,49,50,51]. This binding could be facilitated via an interaction between the PXXP motifs in the proline-rich region and the SH3 domains of the src-family non-receptor tyrosine kinases (e.g., kinase FYN) [52,53,54]. Note that this plasma membrane interaction might play an important role in vesicle-mediated secretion and therefore impact the cell-to-cell spreading of protein Tau. It was proposed that the Tau-FYN interaction may regulate the post-synaptic targeting of FYN, and thereby mediate Aβ-induced excitotoxicity [23]. Additional proposed pathways for cell-to-cell transfer are tunneling nanotubes and trans-synaptic spreading [55,56,57,58]. This spreading process is still ill-defined and it still needs to be proven if spreading of (a) toxic Tau species is sufficient or necessary for the induction of a tauopathy. Guo and colleague published a comprehensive article on this emerging field within Tau biology [59]. Finally, the proline-rich domain of Tau has a role in facilitating the binding of the MT binding region to the MT [60,61].

Tau function is also regulated by several post-translational modifications including phosphorylation, glycosylation, truncation, nitration, isomerisation, acetylation, glycation, ubiquitination, deamidation, methylation, sumoylation and oxidation [38]. Tau phosphorylation has been studied extensively. The protein contains 80 putative serine/threonine and 5 potential tyrosine phosphorylation sites, of which the majority is phosphorylated in vitro, on the 2N/4R (2 N-terminal inserts and 4 amino acid repeat regions) isoform. Tau is phosphorylated by numerous kinases, grouped in 4 different classes [62,63]. More recently, GSK3α, GSK3β, MAPK13 and AMP-activated protein kinase were found to play an actual role in in vivo Tau phosphorylation using different cell lines [64,65]. Tau (de-) phosphorylation is an important factor influencing Tau’s affinity for MT, thereby regulating its role in MT (de-) polymerization. On the other hand, aberrant phosphorylation (so-called hyperphosphorylation) on several epitopes (e.g., Thr181, Thr231, Ser202, Ser205, Ser214, Ser396, Ser404, Ser409, and Ser422), which severely affects Tau’s MT binding capacity and stabilizing properties [66,67,68] can lead to an increased propensity of Tau to subsequently oligomerize and aggregate into paired helical filaments (PHF) and neurofibrillary tangles (NFT) [66]. These NFT are characteristic for a group of neurodegenerative diseases called tauopathies including AD.

The aggregation is due to a redistribution of mainly MT bound to unbound Tau, which facilitates Tau-Tau interactions made possible by 2 hexapeptide motives in repeat regions 2 and 3, which can adapt β-sheet structures [69]. While the repeat domain makes up the PHF core, the N- and C-terminal Tau region form a “coat” around this core [23].

Hyperphosphorylation can also induce pathology through other mechanisms. It can first of all lead to Tau missorting from axons to the somatodendritic compartment, which might cause synaptic dysfunction. Another consequence is affected substrate recognition, which leads to an altered proteasomal degradation [70].

Phosphorylation is, however, only one potential covalent modification Tau can undergo and it should be noted that this modification alone is not sufficient to cause aggregation. Phosphorylation at some sites (e.g., Ser214 and Ser262) in the repeat domain can even protect against aggregation [69]. Thus, it is suggested that phosphorylation might facilitate this process, and therefore serves as an indirect aggregation inducer, and that other factors are involved as well. Indeed, other modifications, and especially truncation, can be equally important for disease development. Tau truncated at Glu391 and Asp421, for example, has been identified as an event following phosphorylation and facilitating Tau filament formation. Tau truncation can even induce neurodegeneration independently of Tau aggregation through the formation of specific Tau fragments [69]. Tau truncation disrupts the paperclip-like structure, thereby promoting Tau aggregation. Tau ubiquitination, on the other hand, is considered a protective strategy of the cell to get rid of toxic Tau intermediates and accumulations of hyperphosphorylated Tau are mainly found in cells with a defective or malfunctioning ubiquitin/proteasome system. The latter can be caused by oxidative stress due to mitochondrial malfunctioning, illustrating the complex cellular pathways involved in the induction of Tau-mediated toxicity. For a more complete overview of Tau post-translational modifications and their consequences on Tau pathology, we refer to [38,71].

The Tau aggregation process itself seems to be a requirement for Tau-induced toxicity and although recent papers are pointing towards the soluble mono- or oligomeric hyperphosphorylated Tau species as being the toxic Tau forms, their relative contribution remains largely unclear. The insoluble aggregated structures are thought to act as protective structures by sequestering the toxic species [72,73,74,75,76].

Numerous Tau mutations, either causing AD or other tauopathies such as frontotemporal dementia and parkinsonism linked to chromosome 17 (FTDP-17), have been documented over the years and can either be missense, silent or causing a deletion. Depending on the mutation’s nature and its gene location, the mutation can directly disturb Tau’s MT binding capacity, thereby increasing Tau’s tendency for aggregation, or indirectly by affecting the 4R:3R ratio by influencing Tau splicing [77,78,79,80,81,82,83,84,85]. Most of these mutations are nicely documented on the “Alzforum” website.

### 2.2. From Complementary Disease Models to AD Diagnostics

Historically, the AD field has been dominated by research supporting Aβ having the main role in pathogenesis. Only after the discovery of several *MAPT* mutations in FTDP-17 did Tau research receive a significant and rightful boost. Indeed, both in vitro and in vivo studies show evidence that Tau is required for Aβ-mediated neurotoxicity [86]. Therefore, only a limited amount of research articles on the pathological aspects of protein Tau using the yeast *S. cerevisiae* as a model organism have been published to date [87,88,89].

**Figure 1:**
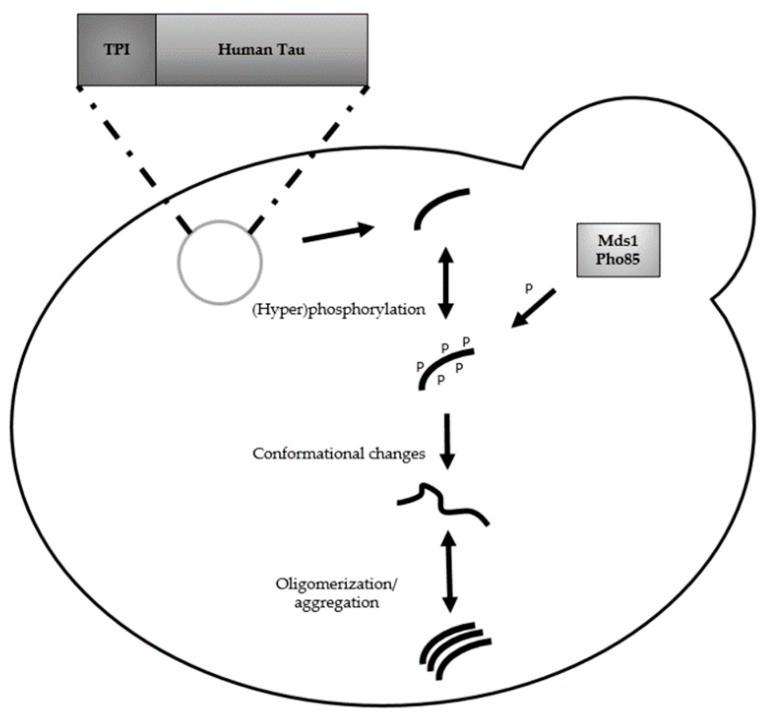
Humanized yeast model expressing human protein Tau: overview of Tau processes and modifications in *S. cerevisiae.* Double arrows indicate a bidirectional/reversible reaction and dashed lines specify the promoter and expressed human *Tau* gene on the plasmid. ‘TPI’; Triosephosphate isomerase promoter, ‘P’; phosphate group.

Figure 1 gives a visual overview of human Tau processes and modifications in *S. cerevisiae*. These studies have already been extensively reviewed, so a brief summary will be given. Upon overexpression in *S. cerevisiae*, Tau becomes hyperphosphorylated and acquires several pathological phospho-epitopes (AD2 (p-Ser396/p-Ser404), AT8 (p-Ser202/p-Ser205), AT270 (p-Thr181), AT180 (p-Thr231/p-Ser235) AT100 (p-Thr212/p-Ser214) and PG5 (p-Ser409)). Moreover, it was possible to detect the disease-relevant conformational epitope recognized by the MC1 antibody.

Pho85 and Mds1 protein kinases, yeast orthologues of human Tau kinases Cdk5 and GSK3β, respectively, were shown to play a key role in modulating Tau phosphorylation. It was suggested that Pho85 may have a direct or indirect inhibitory effect on the activity of Mds1. Upon deletion of Pho85, phosphorylation of Tau was enriched on the AD2 and PG5 epitopes. Accordingly, the MC1-reactive Tau fraction was also higher. Tau aggregation in this *pho85*∆ strain was assessed by measuring the sarkosyl-insoluble Tau (SinT) fraction and it was proposed that Tau epitopes PG5 and AT100 might play a crucial role in the accumulation of SinT aggregates, since these epitopes were especially enriched in the insoluble fraction [87]. The importance of phosphorylation of the PG5 epitope for Tau aggregation was also confirmed in a follow-up study in which aggregation of several Tau mutants was assessed. In addition, PG5 epitope phosphorylation is detrimental for Tau’s MAPT function, illustrated by lack of Tau binding to taxol-stabilized MT from porcine Tubulin in vitro [88,89].

Despite all of this, Tau 2N/4R and 2N/3R expression does not induce an impaired growth phenotype in *S. cerevisiae* [87,88,90]. The latter is not necessarily expected since little attention was paid to the extent of formation of early stage, presumably toxic, soluble (oligomeric) Tau species in these studies. The possibility exists that these oligomeric Tau species are rapidly sequestered in inert aggregates as a cell protection mechanism.

As described above, several other post-translational modifications are expected to contribute to tauopathy development, besides phosphorylation. Therefore, it might be highly interesting to verify if these modifications are also recapitulated in yeast, and if so, to what extend they could offer an explanation for the (lack of) aggregation/toxicity in yeast cells.

On top of that, it was found that oxidative stress and mitochondrial dysfunction, independently of Tau phosphorylation, also strongly induce Tau aggregation in yeast cells [88]. It is also worth mentioning that inducing oxidative stress resulted in Tau dephosphorylation, in accordance with other results obtained from human, rat and mice neuronal cells [91,92]. One potential mechanism is oxidative stress-induced Pin1 activation. Pin1, a peptidyl prolyl *cis*/*trans* isomerase, can then subsequently activate Phosphatase 2a. De Vos and colleagues reported, in accordance with this finding, that a dysfunctional Ess1, Pin1’s yeast orthologue, increases Tau hyperphosphorylation [93].

On the other hand, other studies point out that oxidative stress does not alter Tau phosphorylation or even induces Tau hyperphosphorylation. These studies were performed using a *Dropsophila melanogaster* model and human neuronal cells [94], respectively. The interplay between oxidative stress and Tau phosphorylation, therefore, needs more attention in future studies to further elucidate their relationship.

Tau was also purified, using anion-exchange chromatography, from the previously mentioned *pho85*∆ *S. cerevisiae* strain, maintaining its hyperphosphorylated MC1-reactive state, and could subsequently seed aggregation of wt 2N/4R Tau protein purified from a wt strain in vitro [87]. The possibility of purifying these stable, pathologically-relevant, Tau structures from *S. cerevisiae* cells paved the way for using yeast-purified Tau as an antigen source for mice immunization [95]. This strategy offers a significant benefit over *E. coli* based Tau purifications and antibody generation [76,96,97], since Tau is not post-translationally modified in bacterial cells.

Although oligomerization can be induced by use of, for example, arachidonic acid or heparin, there is no evidence that these artificially formed oligomers/aggregates are the actual toxic species and, therefore, that the produced monoclonal antibodies recognize pathologically relevant Tau species. ADx215, an antibody developed by immunizing mice with 2N/4R Tau purified from a *pho85∆ S. cerevisiae* strain, is capable of detecting both mono- and oligomeric Tau protein [95]. This antibody was recently successfully implemented in a digital enzyme-linked immunosorbent assay (ELISA) platform and able to detect attomolar concentrations of Tau protein, thereby unlocking the potential of Tau as a serum-based AD biomarker [98]. So, over the last decade, yeast has developed from a reliable model organism, merely used to gain more understanding of pathological Tau features such as aggregation and phosphorylation, to a highly suitable platform model for disease-relevant antigen production.

In the NFT of transgenic mice models, α-synuclein can co-localize with Tau and it has been shown that α-synuclein can seed Tau aggregation in vitro and in vivo and even enhance Tau’s toxicity in mice models [99,100,101,102,103]. It is, therefore, clear that the interplay between both proteins is important. Yeast models have been developed that enable the study of both proteins and the resulting effects on toxicity and aggregation [104,105]. Episomal expression of wt and A53T α-synuclein and wt and P301L Tau resulted in increased phosphorylation on the AD2 epitope and Tau aggregation, but no growth-inhibiting effect was detected [105]. The latter was in contrast to a previously reported study [104], where synergistic toxicity was observed upon stable genomic integration of plasmids expressing wt α-synuclein and wt Tau. This is in accordance with increased α-synuclein inclusion formation and Tau phosphorylation/aggregation [105].

### 2.3. Future Perspectives

Baker’s yeast has been of interest to humans since the existence of brewing and bread-making. Since these two activities have been subject to continuous improvement, research on *S. cerevisiae*’s physiology was mainly application-driven. In contrast, focus on the fission yeast *S. pombe* was mainly interested-driven and initial studies were performed to gain more insights in its mating type system and sexual and cell division cycle [106]. Nevertheless, *S. pombe* might offer great potential as a complementary model to study Tau biology since several features such as the cell division machinery, cell polarity and cytoskeleton organization are more closely related to higher eukaryotes compared to *S. cerevisiae* [106,107,108]. This organism could, therefore, be advantageous to study Tau characteristics such as in vivo MT binding, which has not been observed so far in budding yeast models most likely due to critical gene sequence differences. So far, binding to porcine MT has only been shown for yeast extracted Tau [88]. Indeed, fluorescence microscopy studies indicate potential binding of protein Tau to MT in vivo in *S. pombe* cells. Moreover, preliminary data points out that several Tau epitopes also become phosphorylated in *S. pombe* (data not shown). The precise role(s) of each of the fission yeast Tau kinase orthologues remains unresolved.

Heinisch and colleague also proposed several arguments why the milk yeast *K. lactis* could serve as a useful model to study Tau biology, more specifically the effects of energy signaling and oxidative stress on Tau aggregation [109]. *K. lactis* has several advantages over the traditional baker’s yeast model. For example, a respiratory metabolism more resembling that of mammalian cells. Moreover, *K. lactis* did not undergo a whole genome duplication throughout evolution which limits the number of redundant gene functions. This ensures a more easily trackable phenotype upon single gene deletions [110].

Experimental methodologies used in the aforementioned reports, e.g., SinT assay or fluorescence microscopy using a green fluorescent protein (GFP)-tagged Tau protein, are well suited for the study of Tau aggregation, but lack applicability for analysis of Tau oligomerization. Since the current consensus is that oligomeric, rather than aggregated, Tau is the toxic Tau species, neat technologies to study oligomerization could enhance yeast’s value as a model for the study of neurodegenerative diseases such as AD. An example is the use of a split-GFP sensor system. Several split-GFP technologies are described in [111,112,113,114,115].

## 3. Humanized Yeast Models to Study Aβ Biology

### 3.1. Protein Aβ: Structure, Function and Aggregation

Glycoprotein amyloid precursor protein (APP) plays an important role in numerous biological activities, ranging from neuronal development and homeostasis to signaling and intracellular transport [116,117,118,119]. After synthesis in the endoplasmatic reticulum (ER), the protein is subsequently transported from the Golgi apparatus to the plasma membrane where it is cleaved by α- and γ-secretase or β- and γ-secretase following the non-amyloidogenic or amyloidogenic pathway, respectively [120,121]. This cleavage yields several Aβ species with amino acid sequences varying from 40 to 51 amino acids with Aβ_40_ and Aβ_42_ being the final fragments [122,123]. While β-secretase activity is primarily mediated by BACE1, γ-secretase activity actually requires the presence of 4 proteins; Presenilin 1 or 2, Nicastrin, Presenilin enhancer 2 and Anterior pharynx defective 1 (Aph1) [124]. These peptides can then be released in the extracellular space where they can bind to a variety of receptors or they remain associated with the plasma membrane and lipid raft structures [119]. The amyloidogenic pathway is central in the so-called “amyloid cascade hypothesis”, which states that the formed Aβ structures sequentially oligomerize and aggregate thereby causing neurotoxicity and dementia [125]. Aβ peptides can aggregate in different structural forms i.e., soluble oligomers, protofibrils, but also insoluble amyloid fibrils and all of them feature β-sheet structures [126,127]. While the oligomers may spread throughout the brain, the fibrils can further assemble into plaques, which are commonly found in the neocortex of the AD patient brains [128]. However, there is no direct correlation between amyloid plaques and the loss of synapses and neurons in AD patient brains [129,130,131].

In fact, cognitive deficits appear before plaque deposition or the deposition of insoluble amyloid fibrils. Similarly to protein Tau, it is suggested that the Aβ oligomers trigger synapse dysfunction and memory impairment [132,133]. Extracellular receptor-bound Aβ oligomers were proposed to induce neurotoxic effects by causing mitochondrial dysfunction and oxidative stress in neuronal cells, which can cause a massive calcium influx [134]. This can then impair the ability of cells to conduct normal physiological functions [135].

It should be mentioned, however, that it is highly possible that different Aβ forms may contribute to neurodegeneration at different disease stages [135]. A proposed link between Aβ and Tau pathology is the Aβ aggregation-mediated kinase activation, which results in Tau hyperphosphorylation (see above) that in turn results in NFT formation. This is, however, only one possible hypothesis linking these two proteins and their interplay is presumed to be more complex [135]. Other secondary, toxicity inducing, effects of Aβ aggregation are the involvement of the innate immune system and inflammatory responses [136,137,138].

To maintain Aβ protein homeostasis both in the brain and in plasma, production of Aβ is counterbalanced by mechanisms such as proteolytic degradation [139,140,141], active transport via the blood brain barrier [142,143,144,145,146,147] and deposition of Aβ in insoluble aggregates [148,149]. This does not only involve neurons, but also other cells of the neurovascular unit, such as astrocytes [150,151,152]. Disruption of any of these processes might result in neuropathology. Cathepsin B, for example, was identified as a major Aβ-degrading enzyme and its expression level is altered in the brain of AD patients [153].

### 3.2. From Heterologously Expressed APP to Secretory Pathway-Targeted Aβ Peptides

**Figure 2:**
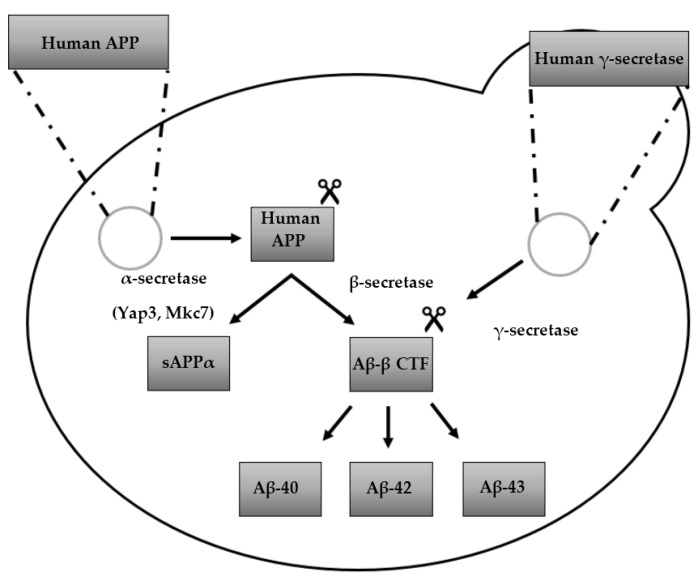
Humanized yeast model expressing human proteins, APP and γ-secretase: overview of Secretase-mediated APP processing and Aβ peptide production. The scissors icon indicates cleavage of the respective proteins.

Since Aβ peptides are generated by Secretase cleavage of APP, modelling of Aβ pathology in yeast cells can be done via a number of approaches. The APP, or the Aβ peptides can be heterologously expressed in yeast. Although there are no orthologues of the human Secretases in yeast present, both α- and β-secretase activity has been reported [154,155], with the yeast proteases Yap3 and Mkc7 suggested to exhibit α-secretase activity [156,157]. γ-secretase activity was successfully reconstituted in *S. cerevisiae* upon combined expression of APP-based substrates and human γ- secretase, resulting in the production of Aβ_40_, Aβ_42_, and Aβ_43_ [158,159] (Figure 2).

As mentioned above, the γ-secretase complex consists of 4 different components and the influence of different combinations of Presenilin and Aph1 proteins on function and substrate specificity of the γ-secretase was tested in a yeast system [160,161]. Although differences were observed, the results were not well in line with parallel studies performed in mammalian cells, which, they reasoned, could be explained by the lack of additional proteins (e.g., GSAP and CD147) that affect γ-secretase function and that are not present in yeast. Moreover, the same group found that Nicastrin can be dispensable for protease activity of double-mutated γ-secretase, e.g., F411Y/S438P [162]. This first group of yeast models, in which pre-Aβ components (i.e., APP-like substrates and Secretases) are expressed, offers the possibility to screen for components and drugs that interfere with Aβ peptide generation and, therefore, have therapeutic potential.

The focus, however, has shifted more towards the expression of the actual Aβ peptides in yeast cells. This has one major benefit since it limits the amount of heterologously expressed proteins in yeast and, therefore, also any potential side reactions which result in non-Aβ pathology associated phenotypes. In this context, the prion-forming capability of *S. cerevisiae* has also been exploited to gain more insight in Aβ’s oligomerization and aggregation capability.

Sup35, for example, is a translation termination factor and has the natural propensity to form self-propagating infectious amyloid aggregates which results in a prion phenotype “[PSI+]” [163].

Bagriantsev and colleague fused the MRF (middle- and release factor domain) of the protein to Aβ_42_ and screened for the ability of an *ade1-14* strain to grown on medium lacking adenine. They showed that fusion of Aβ_42_ to this MRF domain resulted in a similar phenotype as did the N (N-terminal domain) MRF protein, which makes up the entire amino acid sequence of the Sup35 protein. The N-terminal domain is required and sufficient for induction of the prion properties. Aβ_42_ induced oligomerization, resulting in the inability of Sup35 to terminate translation, enabled growth of the *ade1-14* strain on medium lacking adenine by restoring adenine prototrophy [164]. This setup offers a neat in vivo system to screen for modulators of oligomerization. A second, independent study yielded a similar result in that the Aβ_42_-Sup35 fusion was able to form aggregates, although less stable compared to Sup35 aggregates, and restore the [PSI+] phenotype of Sup35 lacking the prion forming domain [165].

To investigate Aβ_42_’s location and interactions, it was fused to GFP. Apart from inducing a growth defect, Aβ_42_ also induced a heat shock response [166]. The latter is in correspondence with data obtained from AD patients that indicate that heat shock protein expression is upregulated in AD as a protective measure [167]. A study on oligomerization/aggregation modifiers using this Aβ_42_-GFP construct suggested that folinic acid might assist in preventing Aβ_42_ misfolding and aggregation [168].

Recently, yeast was also used as a model to screen for rationally designed compounds [169]. More specifically, Thioflavin assays, circular dichroism measurements and transmission electron microscopy were used to assess the efficiency of peptidomimetic inhibitors to inhibit Aβ_42_ aggregation by targeting non-covalent interactions (Table 1). This way, two compounds were able to rescue yeast from Aβ_42_-induced toxicity. Yeast also served as an excellent tool to shed more light on the mechanism of action of the anti-histamine latrepirdine (Dimebon™) [170], which showed promising aggregate clearing activity in vivo (Table 1). The compound was suggested to upregulate the sequestering of aggregated GFP-Aβ_42_ into autophagic-like vesicles which get targeted for degradation. Autophagy plays a crucial role in the removal of aggregated or misfolded proteins, such as Aβ, in neurodegenerative diseases [171,172,173] and impaired clearance of autophagic vesicles is also observed in the brains of AD mice models and patients [171,172,173]. Highly similar results were obtained in other cell and animal models: Steele and colleagues reported that the treatment of cultured mammalian cells with latrepirdine led to enhanced mTOR- and Atg5-dependent autophagy. Moreover, latrepirdine treatment of TgCRND8 transgenic mice was associated with improved learning behavior and with a reduction in accumulation of Aβ_42_ [174].

Finally, a yeast-based screen identified clioquinol and dihydropyrimidine-thiones as compounds being able to ameliorate Aβ toxicity in a synergistic, metal-dependent, way via different mechanisms such as increasing Aβ turnover, restoring vesicle trafficking and oxidative stress protection [175,176] (Table 1). Again, also in transgenic mice models, treatment with clioquinol (analogue) compounds inhibited Aβ accumulation [177] and resulted in a dramatic improvement in learning and memory, accompanied by marked inhibition of AD-like neuropathology [178]. Finally, a study assessed the clinical effect of clioquinol analogue PBT2 using human patient cohorts. Compared to the placebo group, Aβ CSF concentration was reduced upon treating AD patients with PBT2. In addition, some cognitive test results indicated an improvement in AD patients treated with the clioquinol analogue [179]. These research and clinical studies highlight the fact that yeast-based compound screenings are extremely valuable to identify promising molecules that ameliorate Aβ pathology. Secondly, in several cases, the proposed mechanism of action of a compound, based on insights obtained from yeast research, was confirmed by other, often more comprehensive, pathological AD models [174].

**Figure 3:**
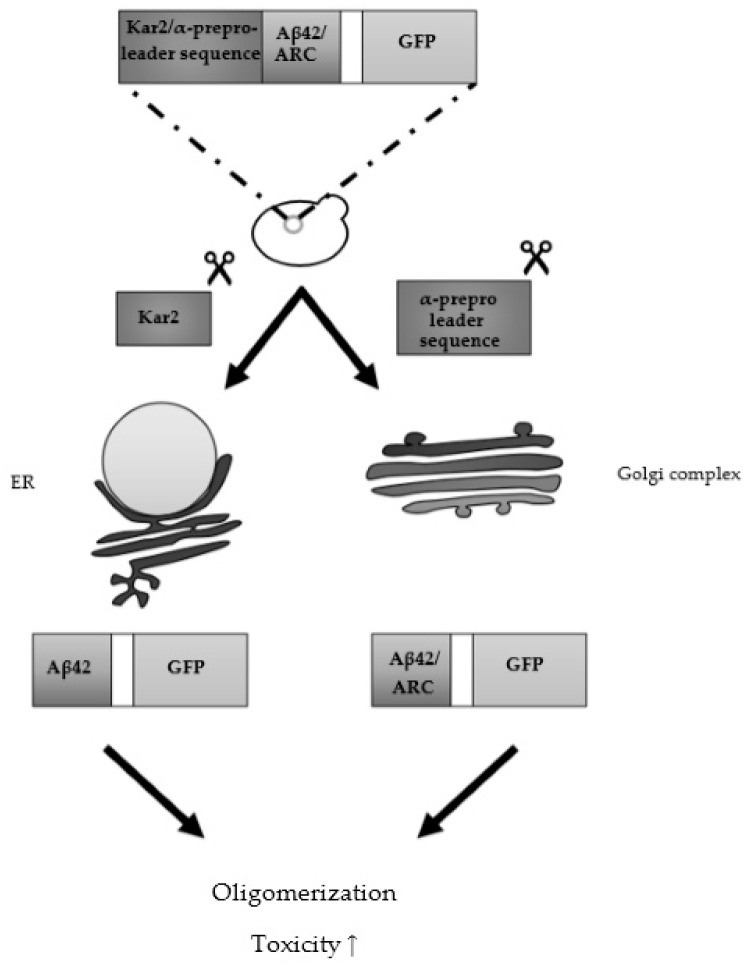
Humanized yeast model expressing GFP-fused Aβ peptides tagged with an endoplasmatic reticulum (ER) or Golgi complex targeting sequence. Treusch and colleagues expressed Aβ_42_ N-terminally tagged with the Kar2 sequence, while D’Angelo and colleagues expressed Aβ_42/ARC_ N-terminally tagged with the α prepro-leader sequence (with and without a C-terminal GFP tag). The scissors icon indicates cleavage of the respective proteins.

Aβ peptides are generated at the plasma membrane and can subsequently be secreted and re-uptaken in the cell and eventually be found in the cytosol, mitochondria, secretory pathway and autophagosomes [180]. To recapitulate Aβ’s multi-compartment trafficking, Treusch and colleagues fused a Kar2 sequence to the N-terminus of Aβ_42_, targeting the peptide to the ER [181] (Figure 3)_._ After cleavage of this sequence, Aβ_42_ is released in the secretory pathway. The presence of a cell wall prevents diffusing of the peptides in the medium, thereby allowing interaction with the plasma membrane and endocytosis. Cell growth was decreased after expression of Aβ_42_ using a multicopy plasmid and galactose-inducible promoter and this in contrast to Aβ_40_. Screening of an overexpression library consisting of >5000 ORFs yielded several suppressors and enhancers of Aβ_42_ toxicity.

PICALM (phosphatidylinositol-binding Clathrin assembly protein), of which Yap1801 and Yap1802 are the yeast homologues [182], was one of the toxicity suppressor hits and is one the most highly validated AD risk factors. The exact role of PICALM in AD is unknown, but it is thought to play a role in APP trafficking [183]. Since Aβ perturbs endocytotic trafficking, it was suggested that PICALM has a role in restoring this process. These findings were backed up by data obtained from rat cortical neurons, in which Aβ-induced cell death was partly prevented upon PICALM expression [181]. A more recent study also reported on the beneficial role of PICALM, since it was able to reduce Aβ_42_ oligomerization [183] (Table 1). In another article [184], the same yeast model was used to study the effects of native Aβ and in addition to previously shown lower growth rate, a reduced respiratory rate and elevated levels of reactive oxygen species (ROS) were exhibited. These are hallmarks of mitochondrial and ubiquitin-proteasome system dysfunction, which also occur in neurons and peripheral tissues of AD patients [185], and nicely illustrate the applicability of such a yeast model to study the role of Aβ in cell stress and damage. In fact, these results were in accordance with findings obtained from a yeast model after prolonged exposure to cytosolic Aβ_42_. Several signs of mitochondrial dysfunction were observed, including increased ROS production, decreased mitochondrial membrane potential and reduced oxygen consumption [184]. A major question that remains is how Aβ peptides actually are taken up in the mitochondria.

In a follow-up study, using a more systems biology approach, the interplay between ER stress and the unfolded protein response (UPR) were studied upon constitutive expression of Aβ_40_ and Aβ_42_ [186]. In comparison to Aβ_40_ which only induced mild stress, Aβ_42_ expression resulted in prolonged high stress and an UPR failing to cope with the unfolded protein load resulting in cellular dysfunction, a shorter chronological lifespan and deregulation of lipid metabolism. These results are highly relevant for other diseases as well, especially cancer and diabetes due to the emerging role of the UPR in these diseases.

Using a similar strategy by fusing the mating type factor α prepro-leader sequence to Aβ, another group also showed that targeting Aβ in the secretory pathway is essential for toxicity in yeast [182] (Figure 3). The researchers tested both native and C-terminally GFP-tagged Aβ_42_ and Aβ_ARC_ and detected aggregate formation and a more profound toxic effect in case of the prepro-Aβ-linker-GFP constructs, especially Aβ_ARC_ for which they measured a decrease in respiratory rate. They also suggested that Hsp104 could play a role in mediating this toxicity by favoring the conversion of large aggregates into smaller oligomeric species. Western blot data showed a decreased protein level in the case of native Aβ peptides, which indicated that the GFP moiety might have a stabilizing effect. These results were not in line with the results published by Treusch and colleagues, since in their research native Aβ_42_ expression resulted in significant toxicity when ending up in the secretory pathway. Another difference was the presumed role of PICALM. In the paper published by D’Angelo and colleagues, deletion of Yap1801 and Yap1802 resulted in a decrease in Aβ-induced toxicity. Upon expression of PICALM, this toxic effect was partly restored. Therefore, more research is necessary to shed light on the actual role of PICALM in Aβ-induced toxicity.

Since it is clear that AD, mitochondrial dysfunction and altered proteostasis are linked to one another, two studies also more closely investigated the interplay between the Pitrilysin Metallopeptidase 1 (PITRM1), an oligopeptide-digesting mitochondrial matrix enzyme, and Aβ. In addition to its role in cleaving the mitochondrial targeting sequence (MTS) of proteins imported across the inner mitochondrial membrane, it also disposes mitochondrial Aβ [187,188,189,190]. However, in a first study, it was shown that the accumulation of Aβ peptides inhibits the activity of Cym1, which is the yeast PITRM1 orthologue, leading to impaired MTS processing and accumulation of precursor proteins [191]. In a second study, the effect of a missense mutation in this enzyme was documented using yeast by modelling the R183Q mutation in the Cym1 protein [192]. This resulted in a reduced Aβ_42_ degradation compared to wt Cym1, suggesting a pathogenic role of this mutated protein, displaying similar behavior as in human beings.

Cenini and colleagues reported that Aβ peptides, especially Aβ_42_, inhibited mitochondrial protein import by affecting an early process step when newly synthesized mitochondrial polypeptides are exposed to the cytosolic environment, rather than affecting mitochondrial membrane potential, TOM and TIM (Translocase of the outer and inner membrane, respectively) or respiratory chain metabolic protein complex composition [193]. These findings are in contrast to the study described earlier in this paragraph [191], in which Aβ peptides indirectly interfered with the processing of imported precursor proteins to the mature and active forms, which is a late step of the mitochondrial import reaction.

Finally, a *C. glabrata* model was used to assess toxicity of extracellular chemically-synthesized Aβ [194] by determining the viable colony count, using a water-based assay. It was shown that Aβ did bind the plasma membrane of *C. glabrata*, but the exact mechanism by which Aβ kills *C. glabrata* remains to be determined. Interestingly, upon oligomerization Aβ loses its toxic effect, while Aβ has a protective function against sodium hydroxide toxicity [195].

## 4. Humanized Yeast Models to Study Frameshift Ubiquitin Mutant UBB^+1^ Biology

Yeast models expressing the frameshift Ubiquitin mutant UBB^+1^ have also been developed. UBB^+1^ accumulation is found in neurons of all AD patients, but absent in those of Parkinson’s disease patients, and co-localizes with the MC1 marker, i.e., NFT [198]. How UBB^+1^ is related to aberrant and phosphorylated Tau protein, both spatially and temporally, still needs to be elucidated [198]. The authors suggested that these mutant proteins may be responsible for the lack of multi-ubiquitination of the hyperphosphorylated Tau fraction found in the NFT [199]. These UBB^+1^ molecules are unable to bind to lysine residues in target molecules, since they lack the COOH-terminal glycine residue in the first repeat region, which is essential for subsequent multi-ubiquitination and activation of the proteasomal machinery [200]. Upon expression of UBB^+1^ in yeast, the protein becomes a substrate of the UPR and accumulated UBB^+1^ impairs the UPR both in yeast and mammalian cells [201,202,203,204]. This results in an accumulation of polyubiquitinated substrates which do not get degraded, partially accomplished by the inhibition of deubiquitinating enzymes [205].

Despite this impairment, no toxicity is observed. By contrast, upon prolonged expression of high levels of UBB^+1^, cell death and mitochondrial dysfunction were observed in neuronal cells and yeast models [203,206,207]. Interestingly to keep in mind here is the fact that UBB^+1^ can be a toxic protein by itself, but it could also act as a potent modifier of toxicity of other neurotoxic proteins, such as Tau and Aβ. Therefore, yeast models combining expression of these proteins in combination with UBB^+1^ could unravel molecular mechanisms important in AD, such as UPS dysfunction and mitochondrial activity [208].

## 5. Studying Prion Characteristics of Aβ and Tau in Yeast

Prions are self-propagating infection protein species. They were first discovered as causative agents in mammalian diseases like Creutzfeldt-Jakob or Scrapie [209,210]. There, a normal protein (PrP^C^) conformationally changes into a malicious and infectious PrP^Sc^ prion protein [211]. Besides these disease-causing prions, a plethora of prions with mostly unknown function has been discovered also in yeast [212,213]. Except for *Podospora anserina*’s [Het-s] prion, most functions of all these prions are unclear and all are toxic, or at least growth-inhibitory, but still are supposed to be beneficial for the survival of cells under stress conditions [214,215,216]. To ensure optimal survival but limit malicious effects, prion formation has to be tightly controlled and carefully balanced. Several factors promote or inhibit prion formation like Hsp104, Hsp70, Sse1, Cur1, Btn2 [217,218,219,220,221]. Interestingly, some of these factors, like Hsp104, promote and inhibit prion formation depending on co-factors and expression levels [222,223].

Many human diseases are described or at least suspected to be prion diseases such as type 2 diabetes mellitus, AD or Huntington’s disease [209,224,225]. Most prions are amyloid and, as its name indicates, Aβ for example stacks β-sheets to form toxic amyloid oligomers which were shown to be transmittable and infectious in mice [226,227,228]. If Aβ is a prion or not is still to be discussed but more and more hints are pointing towards it [227,229]. It is almost accepted that the second key-player in AD, Tau, might be a prion as well. Several studies point towards the MT binding domain of Tau being responsible for aggregation and prionization [230,231]. Several of the yeast models to study AD discussed above [181,182,191,232] are not only used to study the impact of Aβ or Tau on biochemical pathways and on organelles, but also the prion characteristics of these proteins are the focus of research. Evidence for Aβ and Tau being prions were found in mice or other higher eukaryotic model organisms but not in yeast. Tau is hyperphosphorylated and forms aggregates but it is hardly toxic in yeast models [95]. Also, transmission of neither aggregated Tau nor Aβ from affected yeast to healthy strains has been shown so far. But still, there are excellent and robust yeast in vivo techniques to study prion domains and push this field towards greater success. Brachmann and colleagues extended a model developed by Schlumperger and colleagues which makes use of the Ure2 prion system in yeast [233,234]. By replacing promoters of reporter genes by the Ure2 suppressed DAL5 promotor (pDAL5) it is possible to track Ure2-prion strength. If Ure2 is in a non-prion state it binds Gln3, the transcription factor activating pDAL5. When Ure2 forms its prion, [URE3], it releases Gln3 and, thus, induces the reporter gene expression through pDAL5. By replacing Ure2’s own prion domain by any protein domain, one can easily test if it is a prion domain. By making use of different reporter genes it is not only possible to check for a domain to be a prion domain in a black-white manner, like with the URA reporter, but it is also possible to measure the strength of a prion domain by using ADE2 as a reporter. The “redness” of the reporter strain indicates the strength of prion formation and thus the release of Gln3 from unprionized Ure2.

Another technique based on a similar principle is the recently developed yTRAP [235]. Here, suspected prions are fused to a synthetic transcription factor, the synTA. When the protein is soluble and thus not prionized, it allows the synTA to bind the promotor and induce expression of a fluorescent protein, in this case mNeonGreen or mKate2. When aggregated, the transcription factor cannot reach its promotor and the expression of the reporter gene is suppressed. An overview of several traditional and more innovative yeast techniques that play(ed) a crucial role in unravelling Tau and Aβ functions such as protein–protein interaction and prion formation can be found in Table 2.

## 6. Conclusions

It is clear from the above discussed articles that yeast has proven its value in modern AD research. Although *S. cerevisiae* models are most prevalent, it is inspiring to see that alternative models such as *S. pombe* or *C. glabrata* are gaining popularity. The pathobiology of proteins such as Tau and Aβ is robustly recapitulated in yeast. Research using these models has shed more light on the oligomerization/aggregation and prion properties of these proteins, including their role in mitochondrial dysfunction and altered proteostasis, which are two important pathological AD-related cellular processes. This, in combination with the intrinsic benefits of using yeast such as speed and lower costs of research, puts these humanized yeast models in a unique position as a complementary model organism. Therefore, yeast may play a crucial role in overcoming the major future challenges in AD research, including identifying the relationship between all these different pathological AD-related processes.

## Figures and Tables

**Table 1:** Overview of Tau and Aβ toxicity modifiers identified using yeast-based screens.

Protein	Toxicity Modifiers	Description	Other Models	References
Tau	Pin1 (yeast homologue Ess1)	Depletion of Pin1 isomerase activity results in reduced growth of Tau expressing yeast cells.	mouse model	[93,196,197]
Aβ	peptidomimetic inhibitors	Inhibition of Aβ_42_ aggregation by peptidomimetics.	-	[169]
Aβ	latrepirdine (Dimebon™)	Latrepirdine induces autophagy and decreases the intracellular GFP-Aβ_42_ levels in yeast.	Hela cells, mouse model	[170,174]
Aβ	clioquinol	Small molecule screen identified several 8-hydroxyquinolines, including clioquinol, that ameliorate Aβ toxicity.	mouse model, nematode model	[175,176,177,178,179]
Aβ	dihydropyrimidine-thiones	Phenotypic small molecule yeast screen identified dihydropyrimidine-thiones that rescue Aβ-induced toxicity in a metal dependent manner.	nematode model	[176]
Aβ	PICALM (yeast homologues Yap1801, Yap1802)	Screening of overexpression library yielded suppressors and enhancers of Aβ_42_ toxicity, including the PICALM suppressor.	rat cortical neurons	[181,182,183]

**Table 2:** Summary of yeast-based techniques applicable in studies on proteins involved in neurodegenerative diseases.

Technique	Used for	Description
Split-GFP system [111,112,113,114,115,236]	Protein–protein interaction	GFP fluorescence is reconstituted when its two subunits are in close proximity.
Synthetic genetic array [237]	Synthetic lethality	Approach for the systematic construction of double mutants for large-scale mapping of synthetic genetic interactions.
Yeast two-hybrid [238]	Protein–protein interaction	Protein interaction leads to reporter gene expression.
Prion-forming assay [233]	Prion forming	The prion domain of the yeast Ure2 prion is replaced by a potential prion domain of any protein. Reporter gene expression is induced if this domain can complement for the Ure2 prion domain.
Yeast transcriptional reporting aggregating proteins (yTRAP) [235]	Prion forming	High-throughput quantitative prion forming assay. Uses fluorescence as quantifiable reporter.
